# Weight-adjusted waist circumference index and chronic diseases as predictors of depression risk in U.S. adults: a cross-sectional study with mediation analysis

**DOI:** 10.3389/fnut.2025.1568193

**Published:** 2025-07-23

**Authors:** Yali Guo, Meilin Song, Cuixiao Wang

**Affiliations:** ^1^Seventh Medical Center of Chinese PLA General Hospital, Beijing, China; ^2^Department of Hospital Infection Prevention and Control, Hunan University of Medicine General Hospital, Huaihua, Hunan, China; ^3^School of Public Health, Guangxi Medical University, Nanning, Guangxi, China

**Keywords:** depression, chronic diseases, NHANES, mediation analysis, WWI

## Abstract

**Objective:**

This study aimed to examine the association between the weight-adjusted waist circumference index (WWI) and the risk of depression in U.S. adults, as well as the potential mediating roles of chronic diseases (hypertension, diabetes, stroke, and coronary heart disease) in this relationship.

**Methods:**

Data from 7,709 adults aged 20–80 years in the 2017–2023 National Health and Nutrition Examination Survey (NHANES) were analyzed. Logistic regression was used to assess the relationships between WWI (both continuous and categorical) and chronic diseases with depression risk, adjusting for confounders. Subgroup analyses were conducted based on age, sex, race, PIR, education level, marital status, smoking, and drinking status. A restricted cubic spline (RCS) analysis was performed to evaluate the linear relationship between WWI and depression. Mediation analysis was applied to investigate the mediating roles of hypertension, diabetes, and stroke in the WWI-depression relationship.

**Results:**

The final sample consisted of 7,709 adults with a mean age of 50.8 ± 17.4 years, with a depression prevalence of 17% (1,308 cases). Each unit increase in WWI was associated with higher odds of depression in both crude (OR = 1.031, 95% CI: 1.021–1.040) and adjusted models (OR = 1.029, 95% CI: 1.017–1.041). In quartile analysis, higher WWI levels were linked to an increased depression risk compared to the lowest quartile. Subgroup analyses revealed consistent findings, except for differences observed among males and individuals with a high school education or less. RCS analysis showed a linear relationship between WWI and depression risk. Hypertension (OR = 1.038, 95% CI: 1.018–1.058), diabetes (OR = 1.047, 95% CI: 1.021–1.074), and stroke (OR = 1.102, 95% CI: 1.060–1.146) were independently associated with higher depression odds. Mediation analysis indicated that hypertension, stroke, and diabetes mediated 10.3, 2.4, and 10.0% of the WWI-depression relationship, respectively.

**Conclusion:**

Our findings suggest that a higher WWI is independently associated with increased depression risk in U.S. adults. Additionally, chronic diseases such as hypertension, diabetes, and stroke are positively correlated with depression risk. Mediation analysis revealed that these chronic conditions partially mediate the relationship between WWI and depression. These results emphasize the utility of WWI as an anthropometric index for predicting depression risk and highlight the importance of maintaining healthy body composition and managing chronic diseases to prevent depression. Interventions targeting both obesity and chronic disease management may prove effective in mitigating depression risk among adults.

## 1 Introduction

Depression is a widespread mental health condition that poses a significant global public health challenge, in the United States, its prevalence has progressively increased over recent decades (1). Common symptoms of depression include prolonged feelings of sadness, cognitive difficulties, diminished motivation, and, in severe cases, suicidal ideation (2). The origins of depression are multifactorial, encompassing genetic, psychological, and neurobiological influences (3). This disorder not only diminishes the quality of life for those affected but also imposes a considerable burden on families and society, making it a critical concern in both public health and medical fields worldwide (4).

Obesity, characterized by excessive or abnormal accumulation of body fat, poses significant health risks (5). Beyond its association with various physical health issues, obesity is also closely linked to the onset of chronic conditions and an elevated risk of mental health disorders (6). Body mass index (BMI) has traditionally been used as a tool to assess obesity; however, its effectiveness has come under scrutiny in recent years due to its failure to consider variations in muscle mass, bone density, and fat distribution. In comparison, waist circumference (WC) is regarded as a more accurate indicator of obesity-related health risks, given its strong association with abdominal fat (7). More recently, the weight-adjusted waist circumference index (WWI) has been introduced, offering a novel approach that combines weight with an independent measure of central obesity (8). Several studies have shown that WWI provides superior accuracy over BMI when evaluating obesity-related health risks (9, 10).

The Weight-Adjusted Waist Circumference Index (WWI) has been found to be linked to various chronic diseases. One study (11) observed a positive association between WWI and an increased risk of hypertension in a large cohort of Chinese adults. Those in the highest quartile of WWI were found to have a 2.27-fold greater risk of developing hypertension compared to individuals in the lowest quartile, even after adjusting for potential confounders. In a similar cross-sectional study by Zheng et al., higher WWI values were significantly associated with a greater prevalence of type 2 diabetes, independently of BMI and other risk factors (12). Additionally, a prospective study by Hu demonstrated that WWI was a stronger predictor of stroke incidence (13). These results suggest that WWI may provide more comprehensive information than traditional obesity metrics when assessing the risk of chronic diseases. The mechanisms linking WWI to chronic conditions may involve factors such as increased insulin resistance, inflammation, and endothelial dysfunction, which are associated with excessive abdominal fat accumulation (8, 14). Nonetheless, further studies are required to elucidate the exact pathways and assess the clinical relevance of WWI in preventing and managing chronic diseases.

Chronic diseases, particularly hypertension, diabetes, and stroke, have consistently been linked to an increased risk of depression (15). Previous studies have demonstrated that individuals with chronic diseases experience a significantly higher prevalence of depression compared to the general population (16). The relationship between chronic diseases and depression is multifaceted. While chronic diseases have been independently associated with both obesity and depression, their potential mediating role in the relationship between WWI and depression has yet to be systematically explored. Therefore, using data from the National Health and Nutrition Examination Survey (NHANES) 2017–2023, this study aims to: investigate the relationship between WWI, chronic diseases (hypertension, diabetes, coronary heart disease, and stroke), and depression; and examine whether chronic diseases mediate the relationship between WWI and depression, providing a theoretical basis for the prevention and treatment of depression.

## 2 Materials and methods

### 2.1 Study population

Data were derived from the National Health and Nutrition Examination Survey (NHANES) 2017–2023. Of the 27,493 participants initially surveyed, 7,709 adults were included in the final analysis after excluding individuals with missing data on key variables such as race, age, education status, smoking, and others (Supplementary Figure S1).

### 2.2 Assessment of depression

Depression was evaluated using the Patient Health Questionnaire-9 (PHQ-9), a tool designed to assess the frequency of depressive symptoms experienced over the past 2 weeks. Each question is rated on a scale from 0 to 3. Participants with a total score of 10 or higher were categorized as having clinically significant depression (17).

### 2.3 Assessment of weight-adjusted-waist index

Trained medical professionals performed all anthropometric measurements to guarantee their accuracy. To calculate the weight-adjusted waist circumference index (WWI), the waist circumference in centimeters was divided by the square root of the body weight in kilograms (18).

### 2.4 Definition of chronic diseases

Hypertension, diabetes, stroke, and coronary heart disease were classified as either present or absent based on a physician’s diagnosis. Additionally, hypertension was defined as having a systolic blood pressure ≥130 mmHg, a diastolic blood pressure ≥80 mmHg, or the current use of antihypertensive medications (19). Diabetes was considered present if the participant had a fasting plasma glucose ≥7.0 mmol/L, an HbA1c ≥ 6.5%, or was using anti-diabetic medications or insulin (20).

### 2.5 Covariates

Demographic and lifestyle covariates included age, gender, race, poverty-income ratio (PIR), smoking status (defined as having smoked at least 100 cigarettes in a lifetime), alcohol consumption (defined as ever had a drink of any kind of alcohol), education level, and marital status. These variables were obtained through standardized questionnaires or physical measurements.

### 2.6 Statistical analysis

Statistical analyses were conducted using RStudio software (version 4.4.1). Continuous variables are presented as means ± standard deviations and were compared using independent t-tests. Categorical variables are reported as frequencies (percentages) and were assessed using chi-square tests. Logistic regression models were employed to identify influencing factors, including both an unadjusted crude model and a model adjusted for all covariates. Subgroup analyses were performed to explore the association between WWI and depression, stratified by age, gender, race, PIR, education level, marital status, smoking status, alcohol consumption, and hypertension. Furthermore, a restricted cubic spline (RCS) analysis was utilized to examine the relationship between WWI and depression. A two-sided *p*-value of <0.05 was considered statistically significant.

## 3 Results

### 3.1 Baseline characteristics

A total of 7,709 adults aged 20–80 years (mean age: 50.8 ± 17.4 years) were included in the final analysis. Among these, 1,308 participants (17%) were diagnosed with depression. The mean WWI was 11.2 ± 0.9. Regarding chronic diseases, 2,976 participants (38.6%) had hypertension, 1,138 (14.8%) had diabetes, 375 (4.9%) had stroke, and 370 (4.8%) had coronary heart disease. The baseline characteristics of the participants are summarized in Table 1.

**Table 1:** Baseline characteristics of study participants.

Characteristics	Total (*n* = 7,709)	Non-depression (*n* = 6,401)	Depression (*n* = 1,308)	*p* Value
Sex (*n* (%))				
Male	3,341 (43.3)	2,843 (44.4)	498 (38.1)	<0.001
Female	4,368 (56.7)	3,558 (55.6)	810 (61.9)	
Race (*n* (%))				
Non-Hispanic White	3,853 (50.0)	3,203 (50.0)	650 (49.7)	0.844
Non-Hispanic Black and others	3,856 (50.0)	3,198 (50.0)	658 (50.3)	
Marital status (*n* (%))				
Single/divorced/widowed/separated	4,157 (53.9)	3,609 (56.4)	548 (41.9)	<0.001
Married/cohabited	3,552 (46.1)	2,792 (43.6)	760 (58.1)	
Education level (*n* (%))				
Below high school	1,031 (13.4)	797 (12.5)	234 (17.9)	<0.001
High school	1722 (22.3)	1,377 (21.5)	345 (26.4)	
Above high school	4,956 (64.3)	4,227 (66.0)	729 (55.7)	
PIR (*n* (%))				
<2	3,191 (41.4)	2,427 (37.9)	764 (58.4)	<0.001
≥2	4,518 (58.6)	3,974 (62.1)	544 (41.6)	
CHD (*n* (%))				
No	7,339 (95.2)	6,100 (95.3)	1,239 (94.7)	0.417
Yes	370 (4.8)	301 (4.7)	69 (5.3)	
Stroke (*n* (%))				
No	7,334 (95.1)	6,133 (95.8)	1,201 (91.8)	<0.001
Yes	375 (4.9)	268 (4.2)	107 (8.2)	
Hypertension (*n* (%))				
No	4,733 (61.4)	4,003 (62.5)	730 (55.8)	<0.001
Yes	2,976 (38.6)	2,398 (37.5)	578 (44.2)	
Diabetes (*n* (%))				
No	6,571 (85.2)	5,516 (86.2)	1,055 (80.7)	<0.001
Yes	1,138 (14.8)	885 (13.8)	253 (19.3)	
Smoking status (*n* (%))				
No	4,334 (56.2)	3,720 (58.1)	614 (46.9)	<0.001
Yes	3,375 (43.8)	2,681 (41.9)	694 (53.1)	
Alcohol consumption (*n* (%))				
No	563 (7.3)	470 (7.4)	93(7.1)	0.001
Yes	7,146 (92.7)	5,931 (92.6)	1,215 (92.9)	
WWI				
Q1	1928 (25.0)	1,653 (25.8)	275 (21.0)	<0.001
Q2	1927 (25.0)	1,630 (25.5)	297 (22.7)	
Q3	1927 (25.0)	1,599 (25.0)	328 (25.1)	
Q4	1927 (25.0)	1,519 (23.7)	408 (31.2)	
Age (mean (SD))	50.7 (17.4)	51.2 (17.5)	48.7 (16.8)	<0.001
WWI (mean (SD))	11.2 (0.9)	11.1 (0.9)	11.3 (0.9)	<0.001

### 3.2 The association between WWI and depression

**Figure 1:**
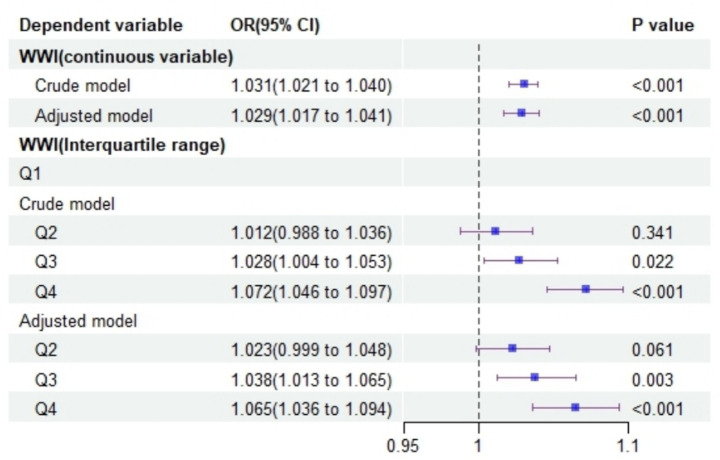
Forest plot illustrating the impact of WWI on depression risk.

Logistic regression analysis was performed with WWI as a continuous independent variable and depression as the dependent variable. Both the crude model (OR = 1.031, 95% CI: 1.021–1.040) and the adjusted model (OR = 1.029, 95% CI: 1.017–1.041) showed that a higher WWI was associated with an increased likelihood of depression, as illustrated in Figure 1.

When WWI was analyzed as a categorical variable in quartiles, participants in the second (Q2) and third (Q3) quartiles had higher odds of depression compared to those in the first quartile (Q1), supporting the previous findings (Figure 1).

**Figure 2:**
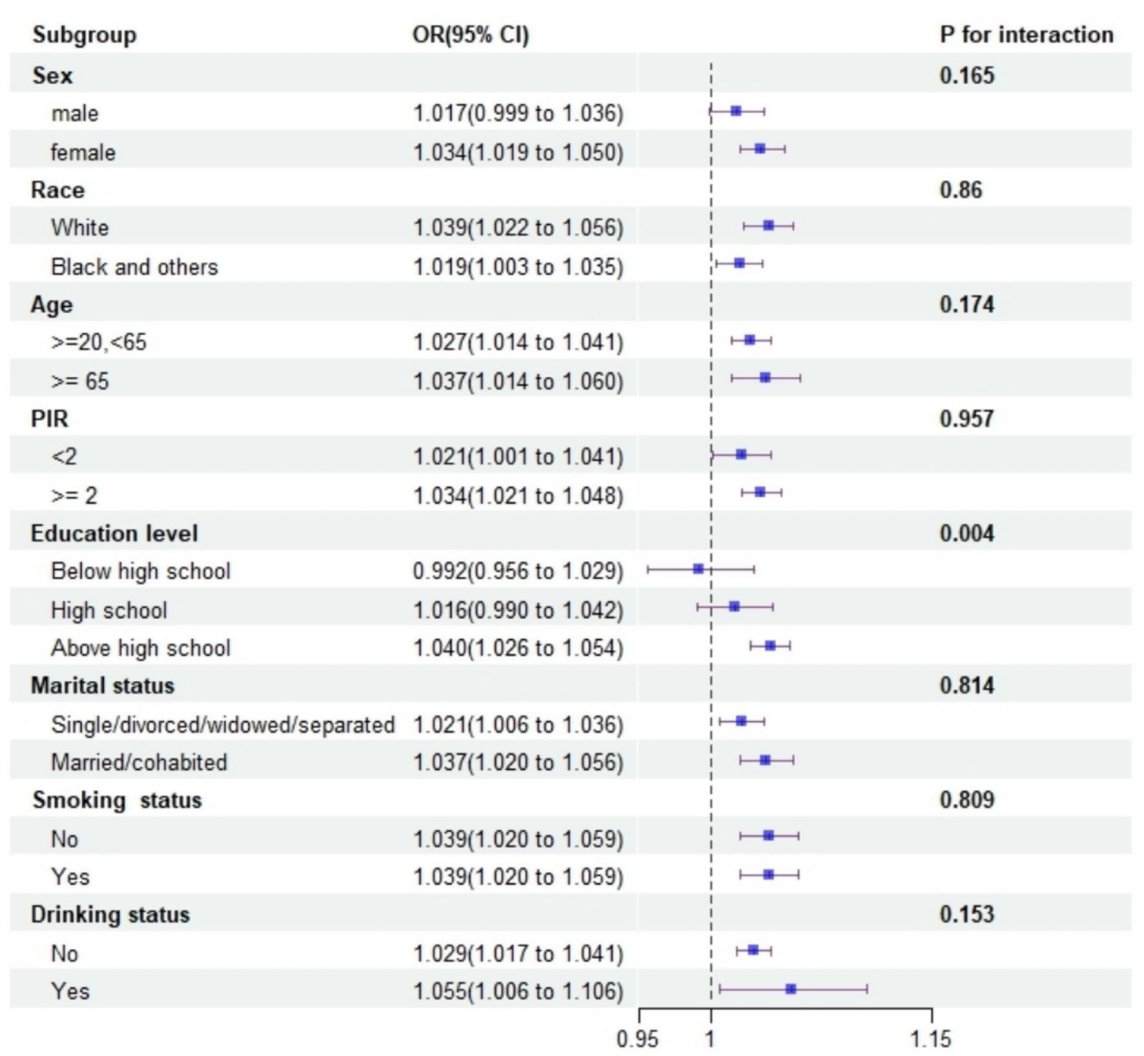
Forest plot of subgroup analyses for the effect of WWI on depression.

**Figure 3:**
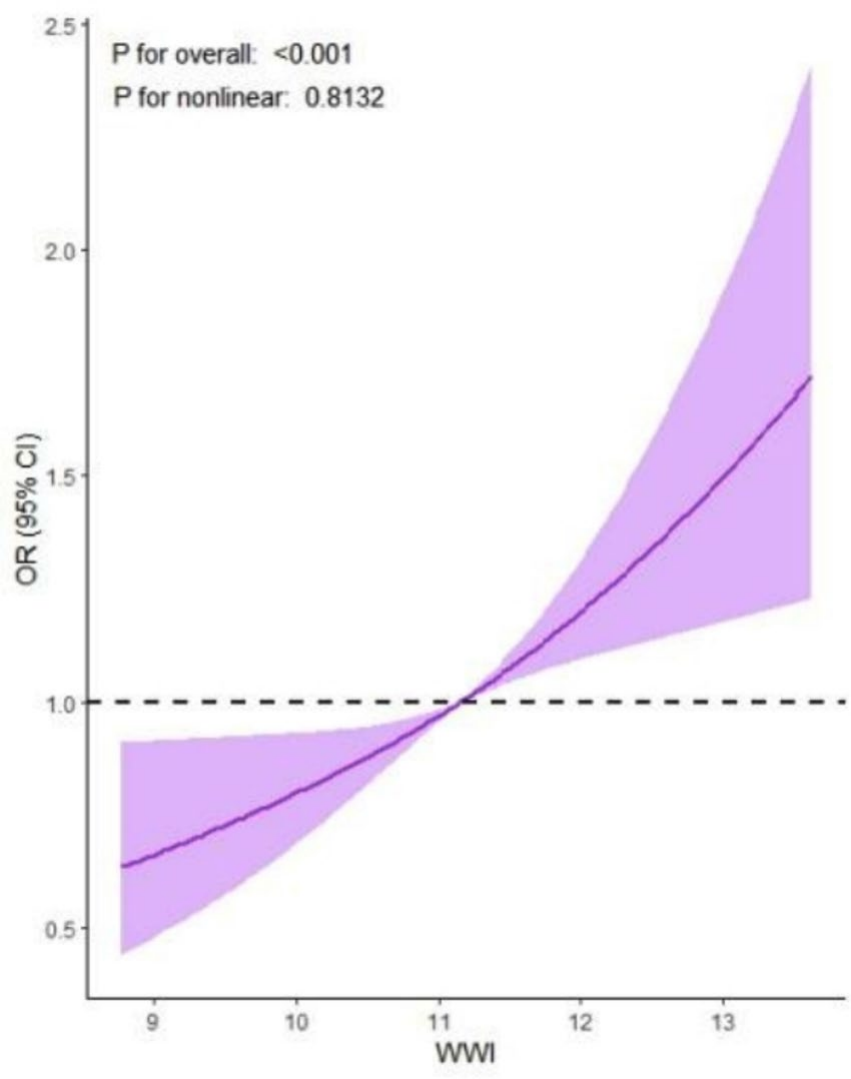
Results of restricted cubic spline analysis illustrating the liner relationship between WWI and depression.

Subgroup analyses consistently supported the main findings, except for heterogeneity observed among males and those with a high school education or less (Figure 2). The restricted cubic spline analysis demonstrated a linear relationship between WWI and depression risk (Figure 3).

### 3.3 The impact of chronic diseases on depression

**Figure 4:**
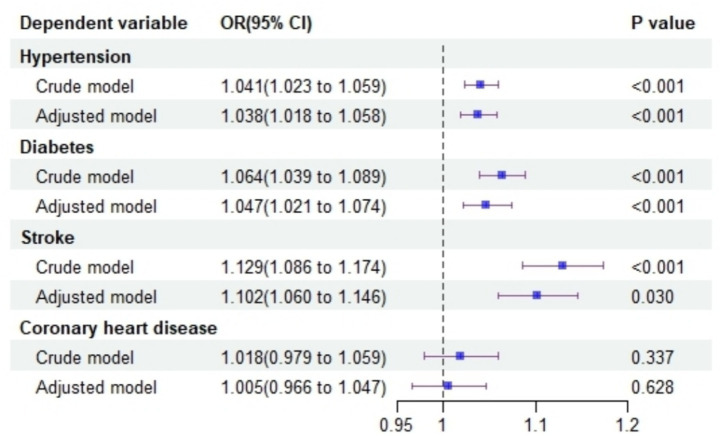
Forest plot illustrates the impact of chronic diseases on depression risk.

Logistic regression analyses were conducted with hypertension, diabetes, stroke, and coronary heart disease as independent variables and depression as the dependent variable. The adjusted models revealed that hypertension (OR = 1.038, 95% CI: 1.018–1.058), diabetes (OR = 1.047, 95% CI: 1.021–1.074), and stroke (OR = 1.102, 95% CI: 1.060–1.146) were significantly associated with an increased likelihood of depression. However, no significant association was observed between coronary heart disease and depression (OR = 1.005, 95% CI: 0.966–1.047), as shown in Figure 4.

### 3.4 Effect of WWI on chronic diseases

**Figure 5:**
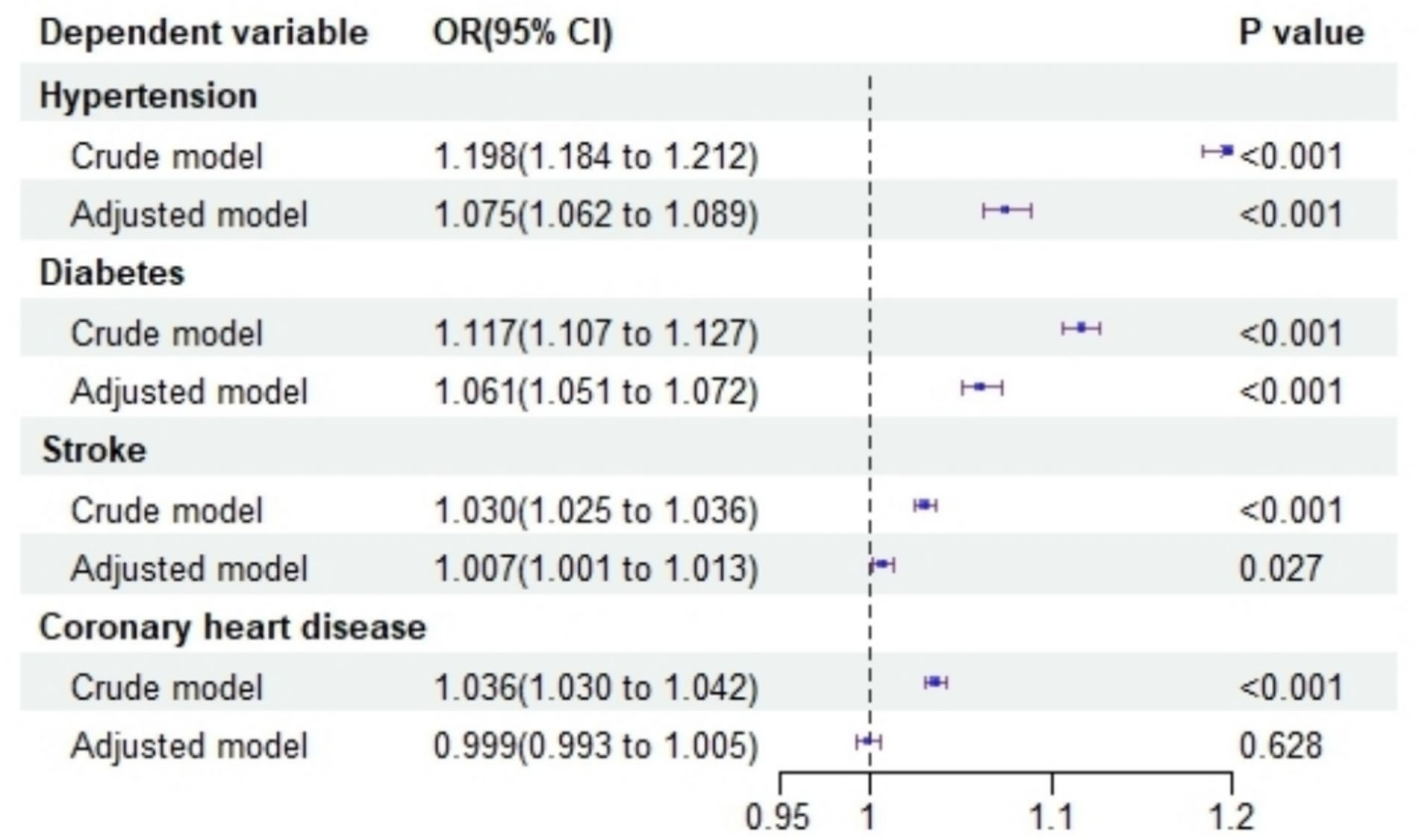
Forest plot illustrating the effect of WWI on chronic diseases.

Logistic regression analysis was performed with WWI as the independent variable and hypertension, diabetes, stroke, and coronary heart disease as dependent variables. The adjusted models indicated that WWI was associated with an increased likelihood of hypertension (OR = 1.075, 95% CI: 1.062–1.089), diabetes (OR = 1.061, 95% CI: 1.051–1.072), and stroke (OR = 1.007, 95% CI: 1.001–1.013). However, no significant effect of WWI on coronary heart disease was observed (OR = 0.999, 95% CI: 0.993–1.005), as shown in Figure 5.

### 3.5 Results of mediation analysis

**Figure 6:**
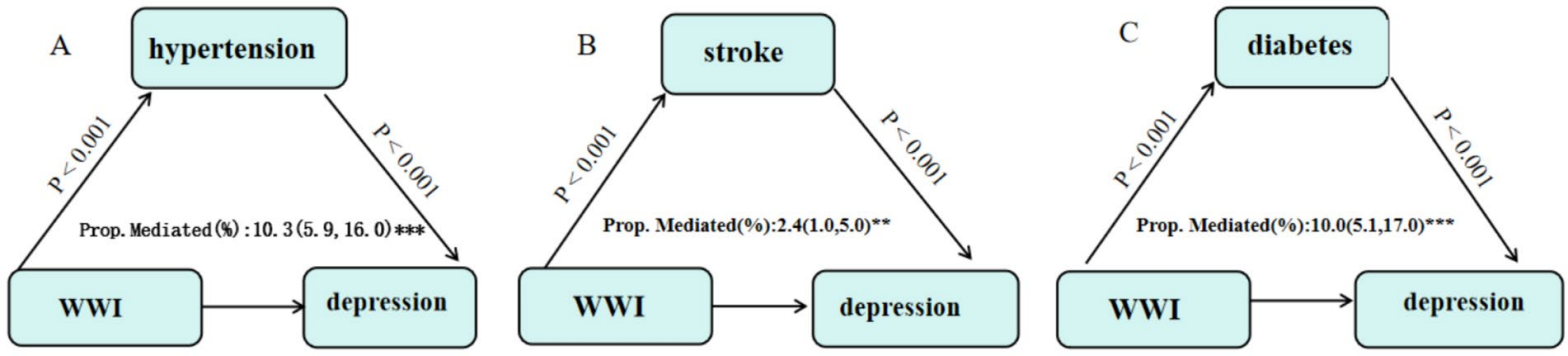
Mediation analysis illustrates the role of hypertension, diabetes, and stroke in the relationship between WWI and depression. **(A)** Hypertension; **(B)** Stroke; **(C)** Diabetes.

Mediation analysis was conducted with WWI as the independent variable, depression as the dependent variable, and hypertension, diabetes, and stroke as mediators. The analysis used the bootstrap method with 5,000 resamples. The results revealed that hypertension mediated 10.3% of the relationship between WWI and depression, stroke mediated 2.4%, and diabetes mediated 10%. These findings suggest that chronic diseases partially mediate the effect of WWI on depression. See Figure 6 for details.

## 4 Discussion

The present study explored the complex relationship between the Weight-Adjusted Waist Circumference Index (WWI), chronic diseases, and depression using data from NHANES 2017–2023. Through comprehensive analysis, including mediation analysis, we examined both the direct effect of WWI on depression and the mediating roles of chronic diseases.

Our results demonstrated a significant positive association between WWI and depression, with both unadjusted (OR = 1.031, 95% CI: 1.021–1.040) and adjusted models (OR = 1.029, 95% CI: 1.017–1.041) showing consistent findings. This relationship exhibited a linear pattern in restricted cubic spline analysis. The underlying mechanisms linking WWI and depression appear to be multifaceted. Primarily, increased central adiposity, as measured by WWI, is associated with chronic low-grade inflammation and altered metabolic function. Adipose tissue produces inflammatory cytokines (such as IL-6 and TNF-*α*) and adipokines, which can cross the blood–brain barrier and influence neurotransmitter systems involved in mood regulation (21). Elevated levels of these inflammatory markers have been shown to interfere with serotonin metabolism and reduce neuroplasticity, potentially contributing to depressive symptoms (22). Additionally, the psychological burden of body image concerns and weight-related stigma associated with central obesity may further exacerbate depressive symptoms through increased psychological stress and altered cortisol regulation (23). Furthermore, our subgroup analyses revealed heterogeneity in the WWI-depression association among males and individuals with lower educational levels. This gender difference might reflect variations in body composition patterns between men and women (24). The educational disparity may indicate differences in health literacy, access to healthcare resources, and lifestyle factors that influence both body composition and mental health (25).

The present study identified significant associations between chronic diseases and depression, particularly for hypertension (OR = 1.038, 95% CI: 1.018–1.058), diabetes (OR = 1.047, 95% CI: 1.021–1.074), and stroke (OR = 1.102, 95% CI: 1.060–1.146). Hypertension has been linked to depression through various biological mechanisms, including vascular damage, neuroendocrine dysfunction, and inflammation. Studies have shown that individuals with hypertension have a higher prevalence of depressive symptoms compared to those with normal blood pressure (26, 27). Similarly, diabetes has been recognized as a risk factor for depression. Chronic stress, functional limitations, and metabolic disturbances associated with diabetes may contribute to the development of depressive symptoms (28). Stroke, a severe cerebrovascular event, has also been associated with an elevated risk of depression. Post-stroke depression is a common neuropsychiatric consequence, affecting approximately one-third of stroke survivors. The neurological damage, disability, and psychosocial challenges following a stroke can contribute to the onset of depressive symptoms (29).

Additionally, our analyses demonstrated that WWI was significantly associated with an increased risk of hypertension (OR = 1.075, 95% CI: 1.062–1.089), diabetes (OR = 1.061, 95% CI: 1.051–1.072), and stroke (OR = 1.007, 95% CI: 1.001–1.013). These findings align with the established role of central adiposity in metabolic disease. Visceral fat, which WWI helps to assess, is metabolically active and produces various factors that contribute to insulin resistance, inflammation, and endothelial dysfunction (30). The absence of association with coronary heart disease in our study, however, contrasts with some previous research and may reflect the complex interplay between adiposity distribution and cardiovascular risk factors.

Importantly, our mediation analysis revealed that chronic diseases partially mediated the relationship between WWI and depression, with hypertension, diabetes, and stroke mediating 10.3% (95% CI: 5.9–16.0%), 10% (95% CI: 5.1–17.0%), and 2.4% (95% CI: 1.0–5.0%) of the total effect, respectively. These findings suggest that central obesity, as measured by WWI, may influence mental health outcomes, such as depression, both directly and indirectly through its impact on chronic disease status. The mediating effect of chronic diseases on the relationship between WWI and depression may be attributed to several potential pathophysiological mechanisms, including inflammation, oxidative stress, and hypothalamic–pituitary–adrenal (HPA) axis dysfunction (31, 32). Elevated WWI values may be associated with increased levels of proinflammatory cytokines and alterations in neuroendocrine signaling pathways, which can impair metabolic function and disrupt emotion regulation processes (33). These findings highlight the complex interplay between obesity, chronic diseases, and mental health, underscoring the need for further research to elucidate the precise biological pathways underlying these associations and to develop targeted interventions for individuals with central obesity and comorbid chronic diseases who are at an increased risk for depression.

The findings of this study have important clinical implications. The observed associations suggest that WWI could serve as a valuable screening tool for identifying individuals at increased risk for both depression and chronic diseases. The significant mediating role of chronic diseases underscores the need for integrated approaches to physical and mental health care, particularly among individuals with central obesity as determined by WWI. Such an integrated approach may involve routine screening for depression in patients with elevated WWI and the implementation of comprehensive lifestyle interventions that address both physical and mental health outcomes simultaneously.

However, several limitations of this study should be acknowledged. First, the cross-sectional design of the NHANES data precludes the establishment of causal relationships between WWI, chronic diseases, and depression. Second, although the PHQ-9 is a validated tool for depression screening, its reliance on self-reported symptoms may introduce subjective bias, potentially leading to an overestimation or underestimation of depression severity. Third, the reliance on self-reported chronic disease status may have introduced recall bias, potentially leading to misclassification of disease status. Fourth, while we controlled for a wide range of potential confounding factors, the possibility of residual confounding cannot be eliminated due to the observational nature of the study.

## 5 Conclusion

In conclusion, this study examined the relationship between WWI, chronic diseases, and depression. The results demonstrated that WWI was significantly associated with the risk of depression, with chronic diseases (hypertension, diabetes, and stroke) partially mediating this relationship. This study provides new insights into the pathways linking central adiposity and mental health and suggests that WWI could serve as a valuable indicator for depression risk assessment. Future lifestyle interventions targeting both WWI and chronic disease management may be effective in preventing and managing depression.

## Data Availability

The original contributions presented in the study are included in the article/Supplementary material, further inquiries can be directed to the corresponding author.

## Supplementary material

SUPPLEMENTARY FIGURE S1 Flow chart of the population included in this study.
