# Supplementary figures and images for: Weight-adjusted waist circumference index and chronic diseases as predictors of depression risk in U.S. adults: a cross-sectional study with mediation analysis
Source: Front Nutr. 2025 Jul 23;12:1568193. doi: 10.3389/fnut.2025.1568193 (PMC12325032; doi:10.3389/fnut.2025.1568193)

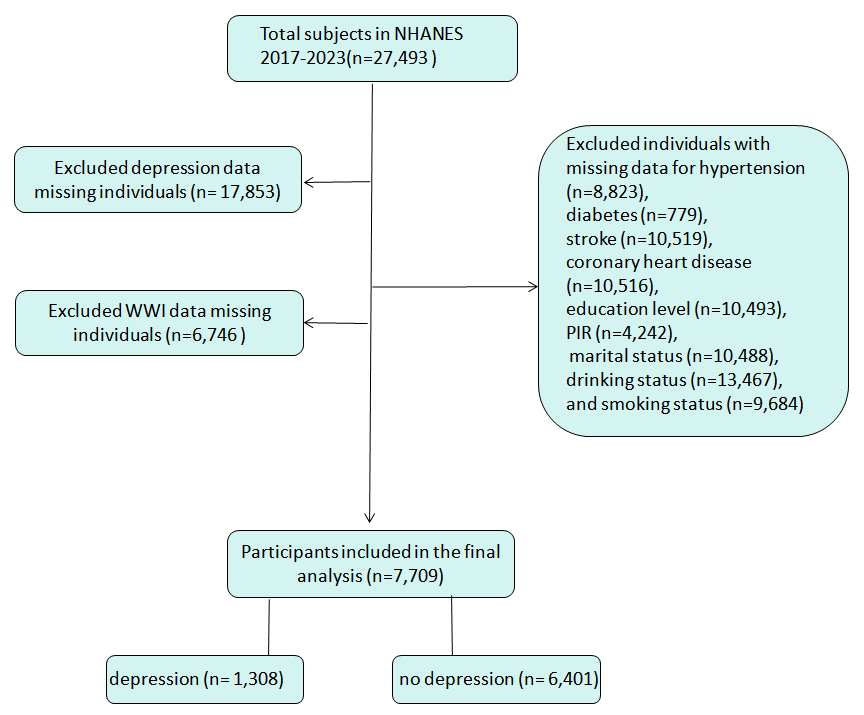

Supplement: SUPPLEMENTARY FIGURE S1 — Flow chart of the population included in this study. [file Image_1.png]
